# Application of the International Classification of Health Interventions for coding interventions in adults with sensorineural hearing loss

**DOI:** 10.1093/jamiaopen/ooaf063

**Published:** 2025-06-27

**Authors:** Faheema Mahomed-Asmail, Ilze Oosthuizen, Catherine Sykes, Soraya Maart, Richard Madden, De Wet Swanepoel, Vinaya Manchaiah

**Affiliations:** Department of Speech-Language Pathology and Audiology, University of Pretoria, Gauteng, 0028, South Africa; Virtual Hearing Lab, Collaborative Initiative between University of Colorado and the University of Pretoria, Aurora, CO, 80045, United States; Department of Speech-Language Pathology and Audiology, University of Pretoria, Gauteng, 0028, South Africa; Virtual Hearing Lab, Collaborative Initiative between University of Colorado and the University of Pretoria, Aurora, CO, 80045, United States; Centre for Disability Policy and Research, University of Sydney, Sydney, 2006, Australia; Division of Physiotherapy, Faculty of Health Sciences, University of Cape Town, Cape Town, 7925, South Africa; Centre for Disability Policy and Research, University of Sydney, Sydney, 2006, Australia; Department of Speech-Language Pathology and Audiology, University of Pretoria, Gauteng, 0028, South Africa; Virtual Hearing Lab, Collaborative Initiative between University of Colorado and the University of Pretoria, Aurora, CO, 80045, United States; Department of Otolaryngology-Head and Neck Surgery, University of Colorado School of Medicine, Aurora, CO, 80045, United States; Department of Speech-Language Pathology and Audiology, University of Pretoria, Gauteng, 0028, South Africa; Virtual Hearing Lab, Collaborative Initiative between University of Colorado and the University of Pretoria, Aurora, CO, 80045, United States; Department of Otolaryngology-Head and Neck Surgery, University of Colorado School of Medicine, Aurora, CO, 80045, United States; UCHealth Hearing and Balance, University of Colorado Hospital, Aurora, CO, 576104, United States; Department of Speech and Hearing, School of Allied Health Sciences, Manipal Academy of Higher Education, Manipal, 576104, India

**Keywords:** hearing health classification, audiology informatics, data aggregation, terminology, International Classification of Health Interventions, World Health Organization

## Abstract

**Objective:**

The International Classification of Health Interventions (ICHI), currently being developed, seeks to span all sectors of the health system. Our objective was to determine the coverage of the ICHI for hearing interventions commonly delivered to adults with sensorineural hearing loss (SNHL).

**Material and Methods:**

A 3-phase content mapping method was used, which included (1) identification of source terms with an expert panel in audiology rehabilitation; (2) 3 coders independently applied the classification to the source terms; and (3) the coders reached a consensus for each intervention and identified reasons for initial discrepancies with options not linked to a specific code were identified.

**Results:**

Nineteen different ICHI Target categories were identified, with 23 different ICHI Action categories and 82% of the means being “Other and unspecified.” There was consensus in codes for 54.3% of source terms, with no ICHI code found for 8.5% of source terms. The greatest number of discrepancies arose from the action, followed by the target. Coding discrepancies occurred as a result of misunderstanding of source terms, the clinical use thereof, and difficulty determining the type of Target.

**Discussion:**

Despite its broad scope, ICHI's current framework has gaps in its coverage of audiological interventions, particularly those related to sensorineural hearing loss. Addressing these gaps is crucial for improving global data standardization and facilitating the development of more targeted hearing health policies.

**Conclusion:**

This study makes an important contribution to the further development and refinement of the classification, specifically in the context of hearing healthcare.

## Introduction

Health terminologies and classifications are essential components of effective health information systems^1^^,^^2^ providing standardized frameworks for collecting, summarizing, analyzing, and contrasting health-related data on a global scale.^3^^,^^4^ The World Health Organization (WHO) maintains a suite of classifications designed to support internationally consistent information on different aspects of health and health management systems.^5^ The 3 core classification systems that make up the WHO Family of International Classifications Network (WHO-FIC) are the International Classification of Diseases (ICD), the International Classification of Functioning, Disability and Health (ICF), and the International Classification of Health Interventions (ICHI). These classifications systematize health and medical concepts such as diagnosis, functioning, and interventions, providing a standardized framework for data collection and exchange.^5^^,^^6^

The ICD and the ICF are widely recognized and have been integrated into both research and clinical practice in the field of hearing healthcare.^7–9^ For example, ICD 10 and 11 are being utilized for documenting procedures within health systems and for billing purposes, with the comprehensive and brief ICF core sets for hearing loss have been developed and used in patient-reported outcome measures.^8^^,^^10^ Initiated in 2007, ICHI was designed to complement existing WHO classifications by providing a comprehensive framework for systematically documenting various health interventions, including those for individuals with disabilities.^5^ ICHI adopts a broad definition of health aligned with the biopsychosocial model, aiming to capture interventions across a wide range of healthcare services, including health promotion, disease prevention, assessment, treatment, rehabilitation, and public health initiatives.^4^

As the newest member of the WHO classification family, ICHI is under active development, aiming to support various health systems initiatives, including statistical analysis, cost comparisons, and the establishment of classification systems in countries where they are currently lacking.^5^^,^^11^ This developmental phase offers an opportunity to test the content of the draft classification and identify additions and changes required to better meet users' needs.^3^^,^^12^ Furthermore, the significance and use of the ICHI extends beyond administrative functions. It aligns with and can be used for monitoring global initiatives such as the Sustainable Development Goals (SDG) and Universal Health Coverage (UCH).^5^ For example, interventions in non-communicable diseases (eg, hearing loss) can be consistently tracked and reported using ICHI codes, making it easier for countries to monitor their progress toward SDG targets. ICHI offers a unified coding system that can also track the availability, quality, and equity of health interventions. This can help in assessing whether essential services are being provided universally and equitably, which is key to UHC.

The ICHI defines a health intervention as “an action performed for, with, or on behalf of a person or population with the aim of assessing, improving, maintaining, promoting, or modifying health, functioning, or health conditions.”^5^ This classification system is structured around 3 main components that include (1) Target—the entity on which the action is performed; (2) Action—the specific action performed by an actor on the Target; and (3) Means—the processes and methods utilized to conduct/execute the action. Each axis consists of coded descriptive categories. The Target axis of ICHI integrates elements from the ICF, including Body Functions, Activities and Participation domains, and Environmental Factors domains, ensuring consistency and compatibility between the 2 classifications. Each intervention in ICHI is assigned a title and a unique 7-digit stem code that denotes the Target, Action, and Means for that specific intervention. Additional information about interventions, such as therapeutic products, assistive products, and medicaments, can be added using extension codes that are linked to the stem codes.

The application of ICHI has been explored across a few healthcare fields, including public health,^13^^,^^14^ medical surgery,^15^ nursing,^3^ and acute stroke care.^16^ For surgical procedures, ICHI has been found generally adequate for compiling international intervention statistics.^15^ In public health, challenges have arisen in classifying separate components within multi-component interventions and operationalizing and coding targets that involve more than one ICHI Target or Action.^13^ In the nursing discipline, it was proposed that new axis categories and intervention codes be added or to modify titles, definitions, and inclusion/exclusion criteria.^3^ For stroke care, a gap in telemedicine was identified.^16^ These investigations have prompted proposed adjustments to address content gaps and eliminate ambiguities, thereby assisting health practitioners in comparing performance of various health systems.^17^

Hearing loss is another area where standardized intervention classifications can improve healthcare delivery. A significant global health issue, hearing loss affects over 1.5 billion people worldwide, with more than 430 million individuals who could benefit from intervention.^18^^,^^19^ By 2050, this figure is expected to rise above 2.5 billion, meaning 1 in every 4 people may experience hearing loss,^19^^,^^20^ largely due to an aging population.^21^ Among older adults, presbycusis, an age-related form of sensorineural hearing loss (SNHL), is particularly prevalent. Resulting from lifelong exposure to auditory damage—such as noise, ototoxic agents, and genetic predisposition—presbycusis is often permanent and associated with declines in quality of life, including increased social isolation,^22^ depression,^23^ and heightened dementia risk.^24^ The ICF Core Set for Hearing Loss is currently utilized as a tool for conducting a comprehensive assessment of the functioning of a person with hearing loss.^7^^,^^12^ While national intervention classification systems may exist, they generally do not cover interventions related to allied health and rehabilitation.^12^

Given these challenges and the ICHI’s ongoing development, this study aimed to assess the draft ICHI classification’s suitability for interventions commonly provided by hearing healthcare professionals, specifically for adults with SNHL. By evaluating ICHI’s current coverage, this study seeks to propose modifications that enhance the classification system’s utility and reliability in the context of hearing health services, ultimately supporting more effective and standardized care.

## Methods

The coverage of hearing interventions for adults with sensorineural hearing loss in the current draft of the ICHI was determined using a content mapping approach, with semantic matching of the source and target terms.^3^ A 3-phase content mapping method was followed, as detailed below.

### Phase 1: identification of the relevant source terminology

As there is currently no specific database of hearing and hearing-related intervention options available,^12^ the research team identified a list of possible interventions using the components identified in a critical review by Laplante-Lévesque et al^25^ as a basis. Subsequently, the list was shared with 10 experts in the field of audiology rehabilitation for adults with SNHL. They were requested to review and provide recommendations to the list of commonly occurring hearing assessments and intervention options to be used as the source terminology during the subsequent phase of the ICHI coding. This iterative process was conducted electronically via email.

To identify relevant ICHI codes, interventions were grouped according to the main phases of the hearing care intervention process, namely (1) screening and diagnostic assessment; (2) technological rehabilitation options (ie, personal acoustic amplification, tinnitus devices, implantable devices, and assistive devices); and (3) rehabilitation intervention (ie, psychosocial rehabilitation solutions, health promotion, prevention, and public health interventions). The applicable format of how these interventions can be administered/delivered was also specified (ie, in clinic/clinician driven; home/community based; telehealth/mHealth; self-administered). The identified codes were linked to the relevant ICF code to support ICHI coding (Table S1).

### Phase 2: independent coding

Following an agreed-upon protocol, 3 coders used the publicly available ICHI coding repository (https://icd.who.int/dev11/l-ichi/en) during January to March 2024 to code the source terminology. The coders included 2 audiologists (F.M.-A. and I.O.) with expertise in both the clinical and rehabilitative aspects of hearing healthcare, alongside a physiotherapist (C.S.) who was an expert coder experienced in ICHI applications across allied health interventions. This diverse team ensured a comprehensive understanding of the coding process while leveraging the specific clinical insights of the audiologists.

The 2 audiologist coders familiarized themselves with the ICHI's structure by studying the ICHI reference guide.^26^ Additionally, cross-checking of the coding of 40% of source terms was conducted by an independent, expert ICHI coder (S.M.). This approach aligns with the method followed by Wübbeler et al^14^ in a recent study on the use of ICHI in public health interventions.

Each intervention in ICHI has a title and a unique 7-digit stem code denoting the Target, Action, and Means for that intervention as defined below:

1. Target—the entity on which the Action is carried out;
2. Action—the deed done by an actor to the Target; and
3. Means—the processes and methods by which the Action is carried out.

There are 3 characters for the Target, 2 characters for the Action, and 2 characters for the Means. For example, the code for “Implantation of cochlear prosthetic device” is CCB.DN.AA, where the Target CCB is “cochlea,” Action DN is “implantation of device,” and Means AA is “open approach” (ie, the specific type of surgical approach is not listed in the stem codes). As the ICHI system does not currently have an automated procedure through a data export option, categorization was done manually by searching for ICHI interventions and procedures using the search box presented on the website. Where a search did not lead to an appropriate coding option, either due to no results presented or unsuitable codes, one of the main axes of ICHI (Target, Action, Means) was used to hand select main and subcategories until a suitable fit was found. For example, “device programming” (main category) was searched for specifically under the axes of action.

The use of telehealth/mHealth during screening, assessment, and rehabilitation interventions was indicated by adding the appropriate extension code(s) to the source terminology. Extension codes expand the detail and granularity of ICHI stem codes; however, they were not integrated to the updated ICHI and thus the extension codes from the Beta version was used (https://mitel.dimi.uniud.it/ichi/).

### Phase 3: discussion and consensus

To ensure the reliability of the coding process, the coders engaged in a consensus-building discussion, reviewing discrepancies between codes. Where possible, a consensus ICHI code was assigned for each source term. The component or axis of discrepancy (ie, Target, Action, Means) or reasons for discrepancies in codes assigned during Phase 2 were recorded (eg, different interpretations of source term meaning). Source terminology not linked to a specific code was captured and suggested coding was provided.

### Data analysis

Based on Phase 2 (independent coding), percentages of 2-way and 3-way intercoder agreements were calculated. In addition, the percentage of source terms for which a consensus ICHI code was found (Phase 3) was calculated. Descriptive qualitative analysis was conducted on the notes recorded during Phases 2 and 3 to explore reasons for discrepancies in codes assigned during Phase 2 as well as other issues that arose during the coding process.

## Results

### Identification of the relevant source terminology

The iterative process to identify the relevant source terminology resulted in 18 items for screening and diagnostic assessment; 40 for technological rehabilitation options; and 36 for rehabilitation intervention.

### Intercoder consensus and agreement

During Phase 2 (independent coding), 100% agreement was found between the 2 audiologist coders. All 3 coders assigned the same ICHI code for 54.3% of source terms (n = 94). The coders found no ICHI code for 8.5% of source terms. For the remaining 37.2%, there was no commonality of results among the coders. For the cross-checked source terms (n = 40), the coders assigned the same code for 15% and no code for 2.5% of source terms. Some examples are given in Table 1.

**Table 1.** Examples of Phase 2 coding results.

Source term	Coding result
Oto-acoustic emissions	Both coders chose ICHI code CTB.AA.ZZ, “Assessment of hearing functions”
Questionnaires for people with hearing loss	Discrepancy on target: One coder chose ICHI code CT2.AC.ZZ, “Test of hearing and vestibular functions” One coder chose ICHI code CTB.AC.ZAA, “Test of hearing functions”
Pure-tone based test	Discrepancy on action: One coder chose ICHI code CTB.AB.ZZ, “Audiometry” One coder chose ICHI code CTB.AC.ZAA, “Test of hearing functions”
Service/repair of personal acoustic amplification	Coders found no appropriate ICHI code

### Analysis of independent coded data

The 3 classification axes can be used to group, analyze, and summarize the coded data. The 94 coded source terms were analyzed by Target, Action, and Means. There were 19 different ICHI Target categories in the coded data. The most common ICHI Target was “Products and technology for communication” (31%), followed by “Hearing functions” (20%) and “Communication” (10%). There were 23 different ICHI Action categories, with the most common categories being “Providing products” (21%), followed by “Assessment” (12%) and “Training” (12%). For 82% of coded source terms, the Means was “Other and unspecified means.” Furthermore, it was noted that a single ICHI code was matched to multiple source terms (see Table 2 and Table S1). Extension codes were only available for telehealth, with specific codes listed, such as XHO2, indicating the provision of intervention directly to a person at a distant location (eg, telephone counselling) or advising or assisting a local provider to perform intervention (fitting or fine-tuning of hearing aid fitting). Although other ICHI extension codes are listed on the current publicly available ICHI, no specific codes are available. For example, the extension code for hearing aids (digital) and batteries is listed, but no code is provided on the current ICHI, and therefore, the extension code from the Beta version can be used, namely XP305.01 (https://mitel.dimi.uniud.it/ichi/).

**Table 2.** Screening and diagnostic intervention services provided to adults with age-related sensorineural hearing loss.

Component	Category	Tools/Intervention	Mode of delivery	ICF code	ICHI code		
					Target	Action	Means
**Screening**	Self-report	Questionnaires for person with hearing loss; Questionnaires for significant others	In-clinic, Community-based, Telehealth	b230 hearing functions, e310 immediate family, e410 individual attitudes of immediate family members	CT2 Hearing and vestibular functions	AC Test	ZZ Unspecified
	Behavioral measures	Speech-in-noise tests	In-clinic, Community-based, Telehealth	b2304 speech discrimination	CTB Hearing functions	AA Assessment	ZZ Unspecified
		Pure tone screening	In-clinic, Community-based, Telehealth	b2300 sound detection	CTB Hearing functions	AB Measurement	ZZ Unspecified
		Tuning fork testing	In-clinic, Community-based	b2300 sound detection	CTB Hearing functions	AA Assessment	ZZ Unspecified
	Objective measures	Otoacoustic emissions	In-clinic, Community-based	b230 hearing functions, s260 structure of inner ear	CTB Hearing functions	AA Assessment	ZZ Unspecified
**Diagnostic assessment**	Case history	General: demographic, relevant medical history	In-clinic, Online form	d240 handling stress and other psychological demands, d760 family relationships, d820 school education, d850 remunerative employment, d910 community life	PZB Whole person	AN Interview	ZZ Unspecified
		Audiological-specific history	In-clinic, Online form	d115 listening, d360 using communication devices and techniques	SE1 Communication	AA Assessment	ZZ Unspecified
		Communication participation assessments (eg, COSI, ICF)	In-clinic, Online form	d310 communicating, d350 conversation, d3503 conversation with 1 person, d3504 conversation with > 1 person, d360 use of communication devices and techniques	SE1 Communication	AC Test	ZZ Unspecified
	Assessment	Otoscopic examination	In-clinic, Community-based, Telehealth	s240 structure of external ear	CZZ Ear and ear functions	AE Inspection	AD Per orifice endoscopic
		Tympanometry	In-clinic, Community-based	s250 structure of middle ear	CBB Tympanic membrane	AB Measurement	AC Per orifice
		Acoustic reflexes	In-clinic, Community-based	s250 structure of middle ear, s110 structure of the brain	MVB Motor reflex functions	AA Assessment	ZZ Unspecified
		Pure tone-based tests	In-clinic, Community-based, Telehealth	b2300 sound detection	CTB Hearing functions	AB Measurement	ZZ Unspecified
		Speech-in-quiet tests; Speech-in-noise tests	In-clinic, Community-based, Telehealth	b2304 speech discrimination, d115 listening	SAD Listening	AA Assessment	ZZ Unspecified
		Diagnostic Otoacoustic emissions	In-clinic, Community-based	b230 hearing functions, s260 structure of inner ear	CTB Hearing functions	AA Assessment	ZZ Unspecified
		Electrophysiological tests	In-clinic, Community-based, Telehealth	s110 structure of the brain, b164 higher cognitive function	CTB Hearing functions	AF Mapping	ZZ Unspecified

### Discrepancies between coders

The greatest number of discrepancies arose from coding the Action axis (26) followed by the Target axis (16). Two key issues were identified as contributing to coding discrepancies. First, the understanding of source terms and the clinical use thereof between the coders. For example, under the category assistive listening devices, the sub-category “remote microphone systems,” the following 2 codes were selected UAF.RD.ZZ and UAF.DP.ZZ. In this example, the action differed, with RD referring to providing products and DP referring to installation of assistive product. A remote microphone is provided by the clinician and not installed by the clinician; however, without a clinical background in hearing healthcare, this would be unknown which resulted in the discrepancy.

Second, there was difficulty in determining the type of Target. It was difficult to decide whether the Target of a source term should be regarded as a provision of an intervention option or an anatomical target with the action linked to the intervention. For example “programming of a sound masking device for tinnitus,” CTK.RD.ZZ and UAF.RD.ZZ were selected by the different coders. As the action RD (providing products) linked to the intervention option, the anatomical target CTK (sensations associated with hearing and vestibular function) was selected over UAF which refers to products and technology for communication. This uncertainty of semantic matching between target concepts in source terms and ICHI codes reflect on the differing professional backgrounds of the coders.

## Discussion

This study aimed to determine the coverage of interventions provided by hearing healthcare professionals to adults with SNHL, utilizing the draft ICHI classification. The main findings with recommendations to enhance the ICHI classification for the hearing healthcare profession will be discussed.

### Classification coverage and representation of hearing healthcare interventions

The low to moderate 3-way intercoder agreement may reflect the ongoing development of the ICHI Classification framework and the variance in familiarity and experience with the classification among the coders. In addition, several coding discrepancies in the Target and Action axes were attributed to differences in coders’ understanding of clinical terminology and the ambiguity in determining the appropriate target for certain interventions. It is noteworthy that the discussions among the 3 coders and the discussion with the coder who performed the cross-checking enhanced understanding of the reasons for coding discrepancies. Differences in understanding or interpretation by coders are likely to influence code selection and increase the incidence of code divergence. These findings highlight the importance of clarity on the details of the reported intervention and the outcome and the need for adjustments for discipline-specific codes and/or extension codes. It can be expected that the reliability of coding will improve once discipline-specific codes and axis categories are more developed.^3^^,^^27^ Machine learning models trained on coding patterns may also reduce discrepancies and errors,^28^ particularly in the Action and Target categories.

Coders were able to assign codes to the majority of the source terms with only 8.5% of source terms being impossible to code, indicating a relatively small coverage gap. However, there were numerous source terms matched to a single ICHI code (see Table 2 and Table S1). In this study, there were 3 ICHI codes that each grouped 4 source terms, one ICHI code that grouped 5 source terms, and one ICHI code that grouped 20 source terms. The ICHI codes onto which several source terms were matched in this study indicate particular areas of the classification that need further refinement to capture specific differences/details of interventions as represented in the source terminology. For example, the code UAF.RD.ZZ (Providing goods for communication) was used for 20 source terms describing a range of personal acoustic amplification options, tinnitus device options, and assistive listening devices. This may be an area needing expansion or adjustment to ensure that the level of detail is sufficient to meet users' needs and to provide adequate distinguishment between intervention source terms. In an evolving classification system like ICHI, it is important to carefully evaluate the grouping of source terms and validate them against empirical data, such as data related to other significant variables like cost. Additionally, incorporating the expertise of relevant stakeholders, including clinicians, researchers, healthcare administrators, and policymakers, is essential to ensure that the classification captures critical distinctions and generates data that can address important informational needs, such as quality monitoring and healthcare funding.^29–31^

### Use of the classification to summarize intervention data

Descriptive analyses of the coded data have demonstrated the use of ICHI’s triaxial structure to summarize data on interventions delivered by hearing health professionals in terms of the types of targets toward which these actions are directed and the types of actions performed. The analysis clearly revealed the most common targets and actions across the 94 source terms. The most frequently coded targets were “Products and technology for communication” (31%) and “Hearing functions” (20%), while the most common actions included “Providing products” (21%) and “Assessment” (12%). This trend suggests that much of hearing healthcare revolves around assessing hearing functions and product provision, indicating where the current classification system may be most effectively applied. Using the results of this analysis would make it possible to explore possible relationships between the interventions’ Target, Action, and Means, and other variables such as patient population characteristics (eg, intervention for the adult vs pediatric population with SNHL).

The findings that 19 and 23 different targets and actions, respectively, were represented across the 94 source terms reflect the complexity and diversity of interventions in hearing health. Although these 2 axes can provide a meaningful basis for discriminating between different interventions, there is a need for more precise coding to accommodate the various aspects of hearing healthcare. There may be potential to further develop the means axis to indicate important distinctions between the methods by which interventions are conducted or delivered, since the majority (82%) of the source terms had an “other and unspecified” means. Additionally, Artificial Intelligence/Machine Learning (AI/ML) tools could be used to identify ambiguous terms and suggest alternative codes, minimizing reliance on “Other and unspecified” classifications.

### Use of results to recommend improvements to ICHI

The coding issues and coverage gaps provide a basis for proposing some recommendations needed to improve the ICHI’s utility and reliability in the field of audiology. This includes reviewing the representation of technological rehabilitation options for adults with SNHL, as 20 of the 40 source terms for technological rehabilitation options were not well catered for. For example, the different source terms listed under personal acoustic amplification options, tinnitus device options, and assistive listening devices were all coded with the same ICHI code, namely UAF.RD.ZZ (see Table S1). It can be proposed that extension codes be identified to distinguish among these different intervention options.

The identification of more precise targets or actions for source terms is recommended. For example, the current ICHI system code for the source term *otoscopic examination* is CZZ.AE.AD, with target CZZ referring to ear and mastoid area unspecified, but CAA can be suggested as the target, referring specifically to the external ear. Combining targets and actions could also be considered to improve clarity and comprehensiveness for particular source terms. The ICHI Reference Guidelines indicate that a combination of targets can be used, for example, SEA (communicating with—receiving spoken messages; current target) and SED (communicating with—receiving nonverbal messages) for the source term *auditory-visual training* to encompass both the verbal and nonverbal communication, thus SEA.PH.ZZ&SED is recommended. Similarly, a combination of actions should be considered, for example for the source term *cochlear implant prosthetic device programming*, combining SM (management of assistive product) and SN (management of internal device) as the clinician will manage and conduct measures of both the internal part (eg, measuring of intra- and extra-cochlear electrode impedances) and the external processor (eg, MAPping procedures); thus, the code would be CCB.SM.AH+CCB.SN.AH.

The use of extension codes, particularly for telehealth, provided additional detail. For example, in the case of the psychosocial rehabilitation component, auditory training (AUE.PH.ZZ), which can be done via telehealth, the following extension code can be used: XH02. However, the analysis also highlighted gaps in the availability of specific extension codes in the publicly available ICHI, necessitating the use of Beta version codes in some instances. For example, adding the extension codes of XXVDB (screening behaviors) and XXCCB (inner ear—cochlear) from the Beta version to the source term *otoacoustic emissions screening* will add to the clarity of this source term and help to distinguish it from the *diagnostic otoacoustic emissions* source term. This highlights the ongoing need for development and refinement within the ICHI system to fully support emerging healthcare technologies and practices.

### Limitations and future research

This study used the ICHI classification that was publicly available between January to March 2024 (https://icd.who.int/dev11/l-ichi/en) and the coding guidelines in the ICHI Reference Guide. Since the ICHI classification is regularly updated, definitions for intervention codes and axis categories are still under development, some codes identified during this study may have thus changed or been relocated since then. It is thus recommended that hearing healthcare experts be involved in identifying issues and gaps with current coding axes and identifying new codes and extension codes. This could assist in ensuring that search phrases in ICHI are more recognizable for coders. Additionally, sophisticated algorithms can be applied through AI tools to learn based on large volumes of healthcare and intervention data with clinical applications.^32^ This could support coders by analyzing clinical terminology and recommending appropriate ICHI codes. Future studies should explore the applicability of ICHI in various healthcare contexts and different countries, including low-resource settings,^33^ which will also support countries in monitoring their progress toward SDG targets and improve comparability among countries. Furthermore, future research should also explore the development and evaluation of AI-driven tools to improve the accuracy and efficiency of ICHI coding.

## Conclusion

ICHI has the potential to serve as a valuable tool in supporting the standardized collection, reporting, and comparison of intervention data across hearing healthcare services. This study makes an important contribution to the further development and refinement of the classification, specifically in the context of hearing healthcare. The results indicate that the ICHI online version has good coverage of audiological interventions; however, a number of specific content gaps and a lack of specificity remain. The ICHI should be used with the ICD and ICF as part of the WHO-FIC network.

## Supplementary Material

ooaf063_Supplementary_Data

## Data Availability

The data underlying this article are available in the article and in its online supplementary material.
