# Supplementary material for: Application of the International Classification of Health Interventions for coding interventions in adults with sensorineural hearing loss
Source: JAMIA Open. 2025 Jun 27;8(3):ooaf063. doi: 10.1093/jamiaopen/ooaf063 (PMC12203548; doi:10.1093/jamiaopen/ooaf063)
Supplement: ooaf063_Supplementary_Data [file ooaf063_supplementary_data.pdf]

Supplementary Table 1. Technological and rehabilitation intervention services provided to adults with age-related sensorineural hearing loss

Component	Category	Tools/Intervention	Mode of Delivery	ICF Code	ICHI Code		
					Target	Action	Means
**Technological rehabilitation intervention services**							
**Technological rehabilitation options**	Personal acoustic amplification: Options	BTE hearing aid, Open-fit hearing aid, ITE hearing aid, CIC hearing aid, RIC hearing aid, CROS hearing aid, BiCROS hearing aid, BC devices, Hearables, PSAPs	In-clinic, Community-based (except CROS, BiCROS); Self (OTCs, Hearables, PSAPs)	d360 using communication devices and techniques, e125 products and technology for communication	UAF (Products and technology for communication)	RD (Providing products)	ZZ (Other and unspecified means)
	Personal acoustic amplification: Programming	Fine-tuning (shaping gain, frequency response, etc.), Fit-related adjustments (physical fit)	In-clinic, Community-based, Telehealth, Self (OTCs)	e2500 sound intensity, e2501 sound quality	UAF (Products and technology for communication)	DP (installation of assistive product)	ZZ (Other and unspecified means)
		Feature activation (pairing, etc.)	In-clinic, Community-based, Telehealth, Self (OTCs)	e2500 sound intensity, e2501 sound quality, e125 product and technology for communication	UAF (Products and technology for communication)	SM (Management of assistive product)	ZZ (Other and unspecified means)
		Service/repair	In-clinic, Community-based	e2500 sound intensity, e2501 sound quality		No code	
		Verification of fitting: REM (including REUG, REAG, REIG, Speechmapping)	In-clinic	e2500 sound intensity, e2501 sound quality	UAF (Products and technology for communication)	AA (Assessment)	ZZ (Other and unspecified means)
		Validation of fitting (aided thresholds, aided speech audiometry)	In-clinic, Community-based, Telehealth, Self	e2500 sound intensity, e2501 sound quality	CTB (Hearing Functions)	AA (Assessment)	ZZ (Other and unspecified means)
		Follow-up self-report measures	In-clinic, Community-based, Telehealth, Self	A range of codes from body functions, sound quality to participation and environment	CT2 (Hearing and vestibular functions)	AC (Test)	ZZ (Other and unspecified means)
	Tinnitus devices: Options	Sound masking device, Sound generator, AC hearing aid options, Sound therapy application	In-clinic, Community-based, Telehealth, Self	e115 products for personal use, d360 using communication devices and techniques, e125 products and technology for communication	UAF (Products and technology for communication)	RD (Providing products)	ZZ (Other and unspecified means)
	Tinnitus devices: Programming	Sound masking device, Sound generator	In-clinic, Community-based, Telehealth, Self	e2500 sound intensity, e2501 sound quality	CTK (Sensations associated with	RD (Providing products)	ZZ (Other and unspecified means)

				hearing and vestibular function)		
	AC hearing aid options	In-clinic, Community-based, Telehealth, Self	e2500 sound intensity, e2501 sound quality	UAF (Products and technology for communication)	DP (installation of assistive product)	ZZ (Other and unspecified means)
	Sound therapy application	In-clinic, Community-based, Telehealth, Self	e2500 sound intensity, e2501 sound quality	CTK (Sensations associated with hearing and vestibular function)	DP (installation of assistive product)	ZZ (Other and unspecified means)
Implantable Devices: Surgical procedures	Cochlear implant prosthetic device	In-hospital	s260 structure of inner ear, d360 using communication devices and techniques, e125 products and technology for communication	CCB (Cochlea)	DN (Implantation of internal device)	ZZ (Other and unspecified means)
	Auditory brainstem implant prosthetic device	In-hospital	s110 structure of brain, d360 using communication devices and techniques, e125 products and technology for communication	AAA (Brain: Encephalon)	DN (Implantation of internal device)	AA (Open approach)
	BC implant prosthetic device, Middle ear implant prosthetic device	In-hospital	s250 structure of middle ear, d360 using communication devices and techniques, e125 products and technology for communication	CBA (Middle Ear)	DN (Implantation of internal device)	AC (Per orifice)
Implantable Devices: Programming	Cochlear implant prosthetic device	In-clinic	e2500 sound intensity, e2501 sound quality	CCB (Cochlea)	SM (Management of assistive product)	AH (external)
				+CCB (Cochlea)	SN(Management of internal device)	AH (external)
	Auditory brainstem implant prosthetic device	In-clinic		No code		

		BC implant prosthetic device, Middle ear implant prosthetic device	In-clinic	e2500 sound intensity, e2501 sound quality	CBA (middle ear)	SM (Management of assistive product)	AC (Per orifice)
	Assistive listening devices	Telecoil, FM systems, Remote microphone systems, Single or multiple loudspeakers, Desktop loudspeaker, Phone accessory, TV accesory	In-clinic, Telehealth, Self	e125 products and technology for communication, e130 products for education, 135 products for employment, e140 technology for sports, recreation	UAF<br><br>(Products and technology for communication)	RD<br><br>(Providing products)	ZZ (Other and unspecified means)
**Rehabilitation intervention services provided to adults with age-related sensorineural hearing loss**							
**Psychosocial rehabilitation options**	Informational counseling	Counseling on the impact, causes, and risk factors of hearing loss & evidence on benefits from treatment	In-clinic, Community-based, Telehealth	d810 informal education, b230 hearing functions	CTB (Hearing functions)	PM (Education)	ZZ (Other and unspecified means)
		Prescription of treatment	In-clinic, Community-based, Telehealth	e1251 assistive products and technology for communication	UAF (Products and technology)	TI (Prescription)	ZZ (Other and unspecified means)
		Counseling on treatment options	In-clinic, Community-based, Telehealth	d360 using communication devices and techniques	CTB (Hearing functions)	PN (Advising)	ZZ (Other and unspecified means)
		Counseling on safe listening	In-clinic, Community-based, Telehealth	d810 informal education, d155 acquiring skills	SAD (Listening)	PM (Education)	ZZ (Other and unspecified means)
		Counseling on hearing conservation	In-clinic, Community-based, Telehealth	d810 informal education, d155 acquiring skills	SAD (Listening)	TM (Environment modification)	ZZ (Other and unspecified means)
		Counseling on importance of healthy hearing across lifespan	In-clinic, Community-based, Telehealth	d810 informal education	CTB (Hearing functions)	PN (Advising)	ZZ (Other and unspecified means)
	Group intervention	Group communication education/Aural Rehabilitation	In-clinic, Community-based, Telehealth	d155 acquiring skills, d360 using communication devices and techniques, d3504 conversing with many people, d825 vocational training	SE1 (Communicatio n)	PM (Education)	ZZ (Other and unspecified means)
	Device usage training		In-clinic, Community-based, Telehealth, Self	d155 acquiring skills, d360 using communication devices and techniques	UAF (Products and technology)	PH (Training)	ZZ (Other and unspecified means)

Auditory perception and speech understanding	Auditory training	In-clinic, Telehealth, Self (app-based)	d1560 auditory perception, b2300 sound detection, b2301 sound discrimination, b2302 sound localization, b2304 speech discrimination, d155 acquiring skills, d825 vocational training	AUE (Perceptual functions)	PH (Training)	ZZ (Other and unspecified means)
	Speech perception training	In-clinic, Telehealth, Self (app-based)	b2304 speech discrimination, d155 acquiring skills	SEA (Receiving spoken message)	PH (Training)	ZZ (Other and unspecified means)
	Auditory-visual training	In-clinic, Telehealth, Self (app-based)	d310 communication spoken message, d315 communication non-verbal messages, d155 acquiring skills	SEA (Receiving spoken message)<br>& SED (Receiving non-verbal)	PH (Training)	ZZ (Other and unspecified means)
	Listening training (top-down, bottom-up)	In-clinic, Telehealth, Self (app-based)	d115 listening, d155 acquiring skills	SAD (Listening)	PH (Training)	ZZ (Other and unspecified means)
	Tinnitus retraining	In-clinic, Telehealth, Self (app-based)	b240 sensations associated with hearing and vestibular function, d810 informal education, d155 acquiring skills	CTK (Sensations associated with hearing and vestibular function)	PH (Training)	ZZ (Other and unspecified means)
Cognitive	Tinnitus, Neuromodulation therapy (e.g., Lenire)	In-clinic, Telehealth (only for tinnitus), Self (app-based, only for tinnitus)	b240 sensations associated with hearing and vestibular function,b164 higher-level cognitive functions, d810 informal education, d155 acquiring skills	CTK (Sensations associated with hearing and vestibular function)	PQ (Psychothera py)	ZZ (Other and unspecified means)
Communication	For person with HL, Assertiveness training (patient empowerment and self-efficacy), Communication partner education	In-clinic, Community-based, Telehealth	d810 informal education, d155 acquiring skills,d310 communication spoken message, d315 communication non-verbal message, e410 attitudes of immediate family, e440 attitudes of personal care providers, e425	SE1 (Communicatio n)	PH (Training)	ZZ (Other and unspecified means)

				attitudes of acquaintances, peers, etc., d825 vocational training			
	Personal	Goal setting	In-clinic, Community-based, Telehealth	b1301 motivation	SE1 (Communication)	TB (Planning)	ZZ (Other and unspecified means)
		Motivational counselling	In-clinic, Community-based, Telehealth	b1301 motivation, d810 informal education	AS1 (Mental)	PP (Counselling)	ZZ (Other and unspecified means)
		Self-efficacy and coping, Family-centred support	In-clinic, Community-based, Telehealth	d155 acquiring skills, d175 solving problems, , e410 attitudes of immediate family, e440 attitudes of personal care providers	SE1 (Communication)	PH (Training)	ZZ (Other and unspecified means)
		Measuring outcomes of intervention	In-clinic, Community-based, Telehealth, Self	Various body functions and structures, participation, environmental codes	CTB (Hearing functions)	AI (Monitoring)	ZZ (Other and unspecified means)
**Health promotion**	Peer support	Peer-mentoring	In-clinic, Community-based, Telehealth, Self	b1301 motivation, e425 attitudes of acquaintances, peers, etc.	UCE (Support from aquintances, peers, colleagues, neighbours and community members)	RB (Practical support)	ZZ (Other and unspecified means)
	Workplace	Noise control measures in public spaces	In-clinic, Community-based	e150 design, construction, building products and technology for public use, d810 informal education, d825 vocational training	No code		
		Vocational Rehabilitation programs	In-clinic, Community-based, Telehealth	e425 attitudes of acquaintances, peers, etc., d825 vocational training	VCB (Workplace safety behaviours)	WI (Restrictions of laws and standards)	ZZ (Other and unspecified means)
		Workplace safety standards	In-clinic, Community-based, Telehealth	e590 labor and employment services, systems, and policies	VCB (Workplace safety behaviours)	VB (Awareness raising)	ZZ (Other and unspecified means)

	Laws and rights	Laws, rights and services for older adults; Laws, rights and services for people with disabilities, including HL	In-clinic, Community-based, Self	e550 legal services, systems and policies, e535 communication services, systems and policies, e590 labor and employment services and policies, e585 educational and training services, systems and policies	UE1 (Services, systems and policies)	VP (Improving access to health services or health-related services)	ZZ (Other and unspecified means)
		Age-friendly community resources	In-clinic, Community-based, Self	e460 societal attitudes, e580 health services, systems and policies, d910 community life	No code		
		Accessibility in residential living (independent living, assistive living and long-term care)	In-clinic, Community-based, Self	e525 housing services, systems and policies, e340 personal care providers and assistants	No code		

*Suggested extension codes as per the Beta version

Note: BTE = behind-the-ear; ITE = in-the-ear; CIC = completely-in-the-canal; RIC = receiver-in- the-canal; CROS = contralateral routing of signal; BiCROS = bilateral contralateral routing of signal; HL = hearing loss
